# Heavy Metal Concentrations in the Spiders *Pirata piraticus* (Clerck, 1757) and *Clubiona phragmitis* (C.L. Koch, 1843) along the Scheldt Estuary (Belgium)

**DOI:** 10.1100/tsw.2002.170

**Published:** 2002-04-10

**Authors:** Catarina Tojal, Frederik Hendrickx, Filip M.G. Tack, Jean-Pierre Maelfait, Nicolas Bogaert, Katrien Willems, Pieter Vernaillen, Johan Mertens, Marc G. Verloo

**Affiliations:** Laboratory of Analytical Chemistry and Applied Ecochemistry, Ghent University, Coupure Links 653, B-9000 Ghent, Belgium; Laboratory of Ecology, Biogeography and Nature Conservation, Ghent University, K.L. Ledeganckstraat 35, B-9000 Ghent, Belgium; Institute of Nature Conservation, Kliniekstraat 25, B-1070 Brussels, Belgium

**Keywords:** heavy metals, bioavailability, Scheldt estuary

## Abstract

Wetland ecosystems may be affected by deposition and accumulation of heavy metals. Metal concentrations in the spiders *Pirata piraticus* and *Clubiona phragmitis* living in marshes along the river Scheldt (Flanders, Belgium) were analyzed. The organisms were sampled on seven sites along a gradient from freshwater to brackish marshes. Except for lead, P. piraticus contained higher metal concentrations than *C. phragmitis*. This is related to physiological and ecological differences between species. No correlation was found between metal concentration in the organisms and soil total concentration.

## INTRODUCTION

As a result of pollution of inland river waters, wetlands may be subjected to deposition and accumulation of heavy metals. Metals can then bioaccumulate in living organisms and proliferate in the food web[1]. Many Flemish rivers and their sediments are more or less contaminated with heavy metals. The Scheldt estuary is a highly industrialized area and, as a consequence, the Scheldt River has been significantly polluted. As a result of frequent inundation of the riverbanks, heavy metals can be deposited and enter the terrestrial ecosystem. High heavy metal concentrations in *Pirata piraticus* living in selected tidal marshes of the Scheldt were already reported[2].

The aim of this study was to assess metal concentrations in arthropods living in wetlands along the Scheldt estuary. The spider distribution pattern along the Scheldt was studied and showed that the species *P. piraticus*, *Clubiona phragmitis,* and *Hypomma fulvum* were abundant along the total length of the estuary from brackish to freshwater marshes[2,3]. This study involves the two similar-sized spider species, *P. piraticus* and *C. phragmitis.* The first is a surface-active spider, while *C. phragmitis* feeds on prey captured on plants.

## MATERIALS AND METHODS

### Study Area and Sampling

The Scheldt River rises in Saint-Quentin in the north of France and flows into the North Sea in The Netherlands after a trajectory of about 300 km in Belgium. The tidal variation extends up to 160 km inland, flooding the river shores twice a day. As a consequence, a gradient can be observed upstream from saline sea water through brackish to fresh water[4]. Spiders were collected at seven different tidal marshes along the Scheldt estuary; those seven are the brackish marshes Saeftinghe and Galgenschoor and the freshwater marshes Galgenweel, Bazel, Notelaar, Kramp, and Konkelschoor (Fig. 1). The Damvallei site is a wetland area along a small tributary of the Scheldt River and was considered a reference area because vegetation was similar to the sampled marshes. In all the marshes, the common reed (*Phragmites australis*) dominates the vegetation. Sampling took place from October to December in 2000. The spiders were collected by hand because pitfall trapping was impossible due to the regular flooding of the marshes.

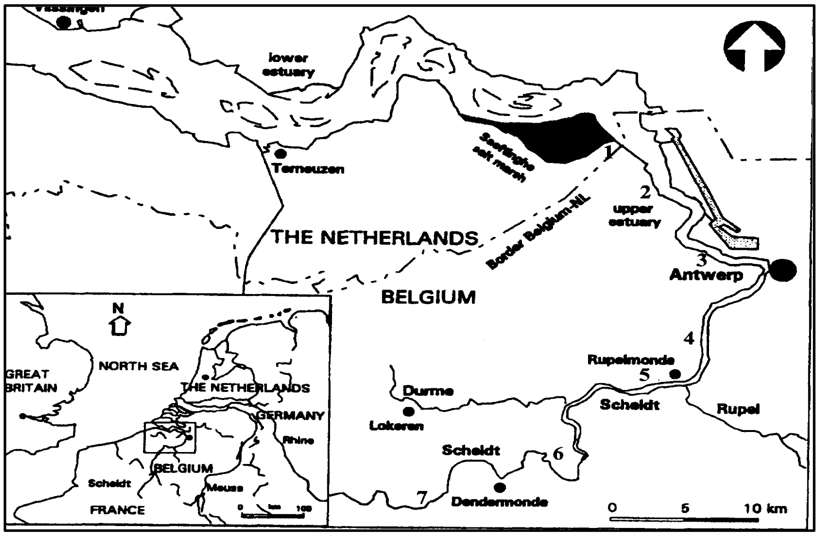

**FIGURE 1.** Sampling sites along the Scheldt River: Saeftinghe (1), Galgenschoor (2), Galgenweel (3), Bazel (4), Notelaar (5), Kramp (6), and Konkelschoor (7) (map modified from[6]).

Moreover, it has been found that the method of pitfall trapping may affect subsequent metal determination[5]. Once transferred to the laboratory, the spiders were stored in a freezer at –10°C. From each of the sampling sites, five spiders were selected for heavy metal analyses. At each of the sampling locations, four soil cores were taken randomly within a distance between 2 and 5 m and transferred to the laboratory. The upper 1 cm of the sediment was sampled for metal analysis. Soil samples were taken in August 2000.

## Heavy Metal Analyses

Individual organisms were subjected to total analysis without further pretreatment. Preliminary tests (data not shown) revealed that washing the spiders briefly with 1% $HNO_3$ prior to analysis did not significantly affect the metal concentration. Wet destruction involving $HNO_3/H_2O_2$ was performed according to Marinussen and Van Der Zee[6]. For analysis of woodlouse, this method yielded 100% recovery compared to a U.S. Environmental Protection Agency method for total analysis of metals in environmental matrices involving microwave destruction in a closed bomb[7]. Soil metal concentrations were determined after aqua regia destruction[8]. The concentrations of copper (Cu), cadmium (Cd), zinc (Zn), and lead (Pb) were measured using flame (Varian SpectrAA-1475) or graphite furnace (Varian SpectrAA-800/GTA-100) atomic absorption spectrometry, depending on the concentration in the sample. The accuracy of chemical analyses was checked using reference samples (bovine muscle [BCR 184], mussel tissue [BCR 278R], and plant material of aquatic origin [BCR 60]).

## Statistical Analysis

Metal concentrations between species and between sites were tested simultaneously with a two-way ANOVA. To test for statistical differences between sites within a species, a one-way ANOVA was performed.

## RESULTS

Higher metal concentrations were found in *P. piraticus* (Table 1) compared to *C. phragmitis*. Only for Pb was the difference between the metal concentrations in the two species not significant ($p > 0.05$).

Differences in Pb and Zn concentrations between the two species of spiders were not dependent on the sampling location ($p > 0.05$). In contrast, Cu and Cd concentrations were significantly dependent on the place of sampling ($p < 0.05$ for Cu, and $p < 0.001$ for Cd). When the two species were considered separately, a significant difference between sampling locations was revealed for Cd, Zn, and Cu in *P. piraticus* and for Cd and Cu in *C. phragmitis*. For *P. piraticus* the highest metal concentrations occurred in the populations of the brackish marshes (Saeftinghe and Galgenschoor), where the metal concentrations were always higher than in the reference site at the Damvallei. For *C. phragmitis* no trend across the salinity gradient was found.

Total metal concentrations in the soils are presented in Table 2. These concentrations were markedly higher than baseline concentrations for surface soils in that region (in mg/kg dry soil: 10–30 for Cu, 0.1–2 for Cd, 30–100 for Zn, and 20–70 for Pb[9]) and higher than those found in the reference site at the Damvallei. Correlations between metal concentrations in the organisms and soil total contents were not significant at the 0.05 level.

**TABLE 1**
**Cu, Cd, Zn, and Pb Concentrations of *P. piraticus* and *C. phragmitis***

| | *P. piraticus* (μg $g^{-1}$ DW) | | | | *C. phragmitis* (μg $g^{-1}$ DW) | | | |
|---|---|---|---|---|---|---|---|---|
| | **Cu** | **Cd** | **Zn** | **Pb** | **Cu** | **Cd** | **Zn** | **Pb** |
| Saeftinghe | 201 ± 43 | 13.0 ± 1.9 | 699 ± 155 | 40 ± 39 | 149 ± 15 | 2.7 ± 1.3 | 517 ± 144 | 39 ± 31 |
| Galgenschoor | 230 ± 74 | 16.1 ± 7.0 | 781 ± 122 | 59 ± 67 | 88 ± 9 | 4.4 ± 1.2 | 431 ± 132 | 25 ± 23 |
| Galgenweel | 157 ± 33 | 10.9 ± 4.2 | 444 ± 65 | 30 ± 28 | 100 ± 39 | 4.5 ± 2.8 | 397 ± 142 | 27 ± 25 |
| Bazel | 173 ± 19 | 12.1 ± 6.4 | 582 ± 75 | 22 ± 5 | a) | a) | a) | a) |
| Notelaar | 163 ± 84 | 7.3 ± 0.7 | 501 ± 29 | 40 ± 53 | 108 ± 22 | 4.9 ± 1.6 | 346 ± 134 | 80 ± 90 |
| Kramp | 127 ± 34 | 14.9 ± 6.3 | 644 ± 86 | 33 ± 19 | 91 ± 14 | 4.1 ± 1.7 | 466 ± 146 | 17 ± 7 |
| Konkelschoor | 179 ± 22 | 9.3 ± 5.1 | 743 ± 274 | 112 ± 172 | 77 ± 26 | 1.2 ± 0.3 | 444 ± 147 | 21 ± 22 |
| Damvallei | 167 ± 62 | 8.1 ± 3.0 | 500 ± 94 | a) | a) | a) | a) | a) |

*Note*: Mean ± standard deviation; n = 5; a) data not available.

**TABLE 2**
**Cu, Cd, Zn, and Pb Total Concentrations in the Soil**

| | Cu (μg $g^{-1}$ DW) | Cd (μg $g^{-1}$ DW) | Zn (μg $g^{-1}$ DW) | Pb (μg $g^{-1}$ DW) |
|---|---|---|---|---|
| Saeftinghe | 33 ± 4 | 2.9 ± 0.3 | 409 ± 40 | 60 ± 8 |
| Galgenschoor | 36 ± 2 | 3.3 ± 0.3 | 385 ± 80 | 64 ± 4 |
| Galgenweel | 84 ± 1 | 6.7 ± 0.3 | 643 ± 85 | 149 ± 5 |
| Bazel | 80 ± 2 | 6.4 ± 0.6 | 669 ± 62 | 133 ± 4 |
| Notelaar | 91 ± 5 | 5.8 ± 0.5 | 689 ± 36 | 130 ± 8 |
| Kramp | 101 ± 10 | 7.0 ± 2.0 | 877 ± 112 | 147 ± 17 |
| Konkelschoor | 80 ± 2 | 4.3 ± 0.4 | 692 ± 55 | 131 ± 4 |
| Damvallei | 20 ± 7 | 1.4 ± 0.6 | 185 ± 55 | 80 ± 17 |

*Note*: Mean ± standard deviation; n= 4.

## DISCUSSION

*P. piraticus* generally exhibited the highest metal concentrations, although Pb was not significantly different between the species. The detection of potential differences for Pb was hampered by the very large variability in the observations, with variation coefficients amounting to 100%.

Physiological and ecological differences between species may account for the different accumulation patterns[2,10,11]. Because the metal concentrations of *P. piraticus* and *C. phragmitis* in the different sampling sites were not correlated, the physiological characteristics alone do not explain the differences. The wolf spider *P. piraticus* lives preferentially on the soil[12], while *C. phragmitis* forages on insects on the reed plants[13]. This difference in foraging behavior results in a different prey composition and, as a consequence, a difference in the amount of heavy metal ingestion. Although the diet of these two species is not known in detail, a difference is that while *P. piraticus* feeds mostly on soil insects, *C. phragmitis* also has access to flying insects that tend to have a lower concentration of metals than the ones who live on the soil[10]. This agrees with our results that metal concentrations were lower in *C. phragmitis*.

Metal concentrations in a particular species depend at least as much on ecological aspects as on the physical and chemical characteristics of the soil. Considering the importance of spiders in the food chain, more studies are needed to clarify the exact diet of these species and in what way their preys accumulate heavy metals. This information is needed to determine more precisely the pathways by which metals accumulated in soils are transferred into the food chain.
